# Design of high gain base station antenna array for mm-wave cellular communication systems

**DOI:** 10.1038/s41598-023-31728-z

**Published:** 2023-03-25

**Authors:** Daniyal Ali Sehrai, Jalal Khan, Mujeeb Abdullah, Muhammad Asif, Mohammad Alibakhshikenari, Bal Virdee, Wahab Ali Shah, Salahuddin Khan, Muhammad Ibrar, Saeedullah Jan, Amjad Ullah, Francisco Falcone

**Affiliations:** 1 Department of Electrical Engineering, University of Oviedo, 33203 Gijon, Spain; 2 Telecommunication Engineering Department, University of Engineering and Technology, Mardan, 23200 Pakistan; 3 College of Aeronautical Engineering, National University of Sciences and Technology, Risalpur (Campus), Nowshera, 24100 Pakistan; 4 Electrical Engineering Department, University of Science and Technology, Bannu, 23200 Pakistan; 5 Department of Signal Theory and Communications, Universidad Carlos III de Madrid, 28911 Leganes, Madrid, Spain; 6 Center for Communications Technology, London Metropolitan University, London, UK; 7 Department of Electrical Engineering, Namal University, Mianwali, 42250 Pakistan; 8 College of Engineering, King Saud University, P.O.Box 800, Riyadh, 11421 Saudi Arabia; 9 Department of Physics, Islamia College, Peshawar, 25000 Pakistan; 10 Department of Electrical Engineering, University of Engineering and Technology, Peshawar, 25000 Pakistan; 11 Department of Electric, Electronic and Communication Engineering and the Institute of Smart Cities, Public University of Navarre, 31006 Pamplona, Spain; 12 Tecnologico de Monterrey, School of Engineering and Sciences, 64849 Monterrey, Mexico

**Keywords:** Engineering, Electrical and electronic engineering

## Abstract

Millimeter wave (mm-Wave) wireless communication systems require high gain antennas to overcome path loss effects and thereby enhance system coverage. This paper presents the design and analysis of an antenna array for high gain performance of future mm-wave 5G communication systems. The proposed antenna is based on planar microstrip technology and fabricated on 0.254 mm thick dielectric substrate (Rogers-5880) having a relative permittivity of 2.2 and loss tangent of 0.0009. The single radiating element used to construct the antenna array is a microstrip patch that has a configuration resembling a two-pronged fork. The single radiator has a realized gain of 7.6 dBi. To achieve the gain required by 5G base stations, a 64-element array antenna design is proposed which has a bore side gain of 21.2 dBi at 37.2 GHz. The 8 × 8, 8 × 16, and 8 × 32 antenna array designs described here were simulated and optimized using CST Microwave Studio, which is a 3D full-wave electromagnetic solver. The overall characteristics of the array in terms of reflection-coefficient and radiation patterns makes the proposed design suitable for mm-Wave 5G and other communication systems.

## Introduction

The fifth generation (5G) technology standard for broadband cellular networks is needed to accommodate the exponential growth in the wireless data traffic driven by the proliferation of smart devices and live streaming. With advancement in technology, it is anticipated that the 5G wireless system will need to cope with a huge amount of data traffic resulting from high-resolution video streaming, tactile Internet, IoT based remote monitoring, real-time control applications and connected devices and vehicles. Billions of devices will be connected to the global IP network which will be a challenge and will require a lot of bandwidth^1^. At the present the overflow of data has primarily been attributed to streaming video but the emergence of new unforeseen applications like holographic tv will introduce even more demand on bandwidth^2^. To support good user experience, the 5G network will also have to reach unique levels of flexibility and intelligence; spectrum regulation will need to be reviewed and enhanced, energy and cost efficiencies will become even more serious concerns^3,4^.

The current 5G wireless communication system is not equipped to meet this explosive growth in traffic demand hence the need for 5G systems to operate at millimeter-waves to acquire much larger bandwidth to accommodate the intense data traffic. The frequency bands below 20 GHz are highly congested, so a shift to higher part of the frequency spectrum is inevitable^5^. The prominent frequency bands for the 5G mm-Wave based cellular communication are 24–28 GHz, 37–40 GHz, and 64–71 GHz^6,7^. This will inevitably raise new challenges^8–10^ including the free space path loss and hardware impairments. The amount of array gain required to compensate free space path loss becomes higher with the increase in frequency and the impact of atmospheric absorption due to carbon dioxide, oxygen and rain/fog/snow attenuation will impact significantly on service coverage compared to existing broadband mobile systems. Consequently, the number of antennas required needs to be increased. This increases the associated electronics with each antenna elements making the overall array very expensive compared to its lower frequency counterpart^11^.

A lot of work has been done recently in developing antennas for 5G wireless systems covering the frequency band 37–40 GHz^5,12–16^. At this frequency band the atmospheric losses are effectively minimal^17^. This should to some extent ease the design of 5G mm-Wave systems to achieve specifications of high bandwidth and data rate requirements. Reported in^5^ is an antenna operating at 38 GHz, i.e., one of the 5G, which could handle the atmospheric attenuations challenge at mm-wave transmission. The peak gain of the antenna is 10 dBi. An array antenna comprising four elements is reported in^12^ which operates at the 38 GHz band. This antenna array is shown to have a gain of greater than 12 dBi, which is sufficient for the 5G mobile systems. Similarly, the antenna presented in^13^ operates over the 5G mm-Wave band, i.e., 37–40 GHz. The array configuration exhibits gain of up to 12 dBi. Antenna array in^14^ covering the 37–39 GHz band has a maximum gain of 8.81 dBi. The concept of metasurface would be helpful for the design of base station antennas operating over 5G mm-Wave spectrum^15–17^. Metasurface is used to enhance the antenna’s performances in^18^ but the maximum gain is limited to 8.91 dBi. The antenna presented in^19^ can cover the band from 37.1 to 38.1 GHz, but no gain enhancement is implemented to handle the atmosphere attenuations challenge. Likewise, an array structure is implemented in^24^ for the similar antenna element presented in^18^ to improve the gain. But still, maximum gain of nearly 12 dBi is achieved which is not sufficient for base station communication. Thus, the antenna element design presented in^18,24^ is considered in this work to develop such an array structure which could provide enough gain for the base station communication.

The antennas mentioned above are dedicated for the 5G mobile devices but cannot be adopted for base station applications because of their low gain. Consequently, in this work, we propose a novel antenna array suitable for 5G mm-wave base station applications. Each radiating structure in the antenna array consists of a 2 × 2 array. This configuration is used to boost the overall gain of the array at the mm-wave band. The gain of the proposed 8 × 32 antenna array is more than 20 dBi at 37.2 GHz, which is suitable for application in future 5G mm-Wave base stations.

## Design methodology

### Antenna array evolution

**Figure 1:**
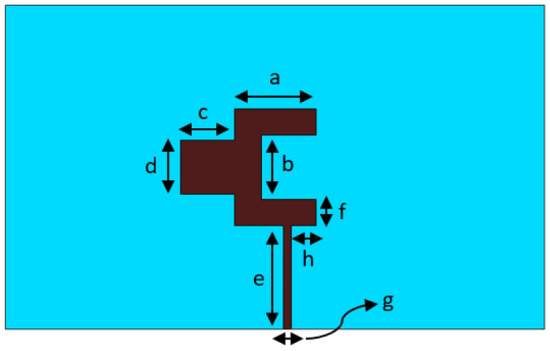
Geometry of the proposed antenna element used in the antenna arrays.

The geometry of the antenna used in the design the 5G mm-Wave arrays, i.e., 8 × 8, 8 × 16, and 8 × 32, is shown in Fig. 1. The antenna resembles a two-pronged fork that is excited with a microstrip feedline that is edge connected to the U-shaped arm of the fork^18,24^. This antenna configuration was chosen for its impedance matching characteristic compared to the conventional rectangular patch antenna^24^. However, it has a smaller effective aperture size than a conventional rectangular patch antenna operating at the same center frequency and hence a lower gain. The antenna was designed to resonate at 37.2 GHz. The antenna is constructed on a Rogers 5880 dielectric substrate with a relative permittivity of 2.2, a thickness of 0.254 mm, and loss tangent of 0.0009. The size of the substrate used is 10 × 6 mm^2^. The dimensions of the antenna are listed in Table 1.

**Table 1:** Dimensions of the antenna parameters.

a	1.5 mm	f	0.5 mm
b	1.2 mm	g	0.15 mm
c	1.0 mm	h	0.46 mm
d	1.0 mm	–	–
e	1.9 mm	–	–

**Figure 2:**
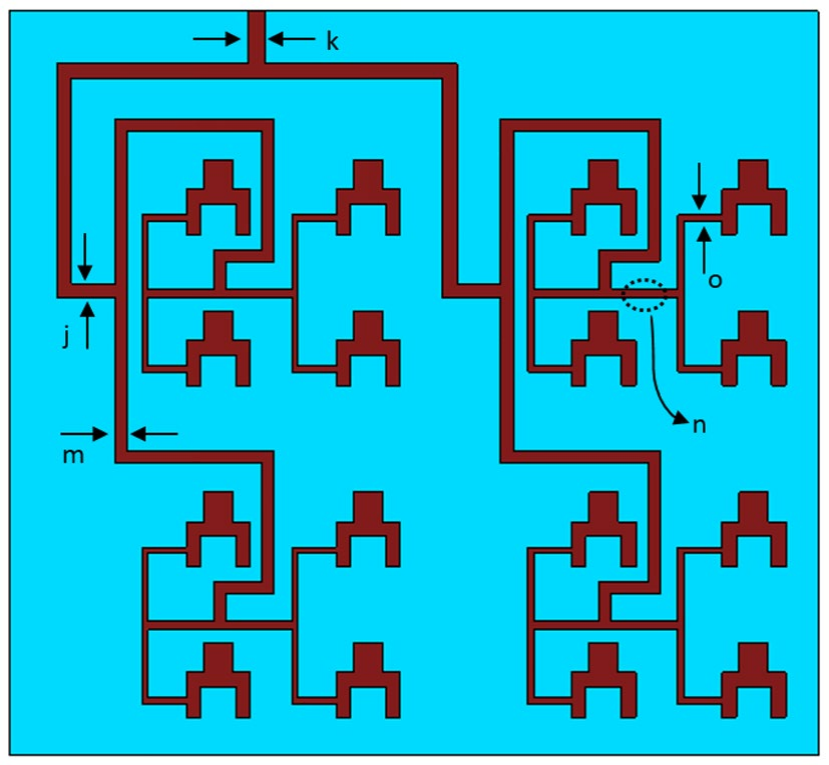
Geometry of the proposed 8 × 8 antenna array.

The proposed antenna was used to implement an 8 × 8 antenna array, shown in Fig. 2, which consist of 16 interconnected antennas using power dividers. The array is excited though a 50-Ω feed network. In the design the distance between the radiating elements was made a multiple of 0.5λ to mitigate mutual coupling among the radiating elements. The spacing between the elements is optimized to obtain the best results. The length of the feedlines is made such that there is phase coherency and power equity at the individual antennas. This is important for preventing the erosion of the gain and radiation characteristics of the array. The dimensions of the other parameters annotated in Fig. 2 are given in Table 2. The overall size of the 8 × 8 array is 25.5 × 27.5 mm^2^.

**Table 2:** Dimensions of the proposed 8 × 8 and 8 × 16 antenna parameters.

k	0.6 mm	p	0.7 mm
j	0.5 mm	g	0.15 mm
m	0.41 mm	h	0.46 mm
n	0.3 mm	–	–
o	0.19 mm	–	–

**Figure 3:**
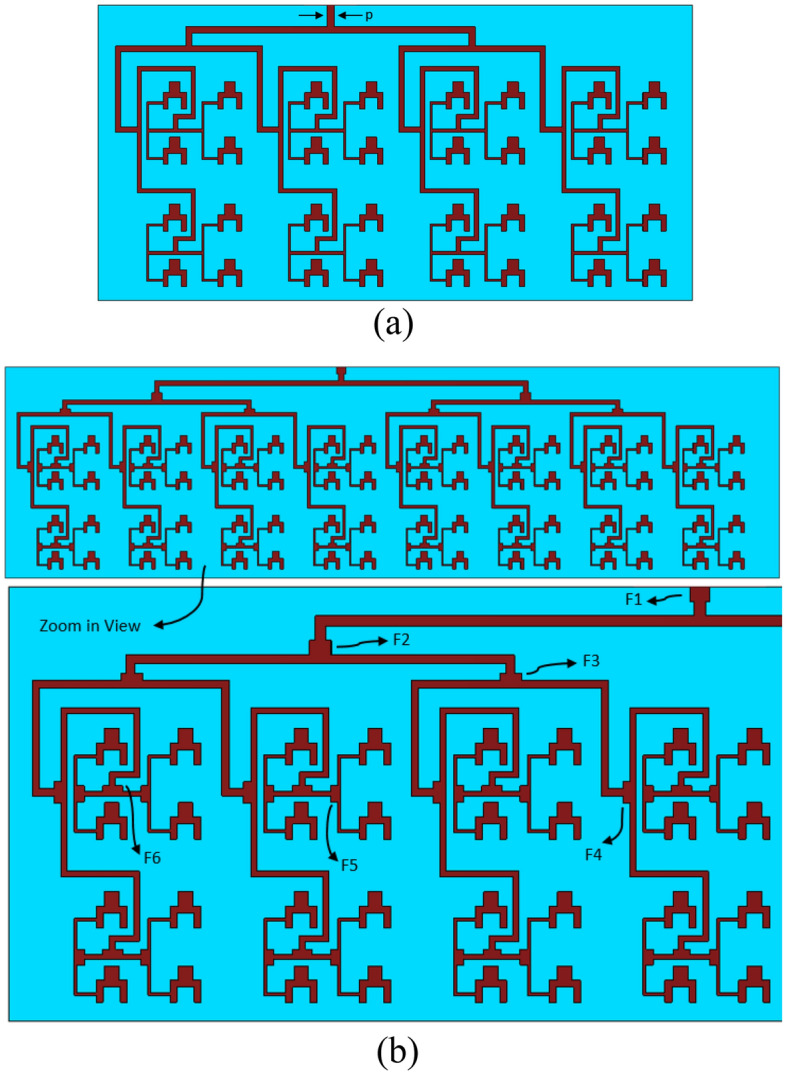
Geometry of the proposed antenna array, (**a**) 8 × 16, and (**b**) 8 × 32.

The 8 × 8 array was extended to 8 × 16 array, which consisted of 32 antenna elements. This was achieved by using a power divider to split the input signal into two equal phase output signals that are applied to the two 8 × 8 array, as shown in Fig. 3a. The overall size of the 8 × 16 array is 27.5 × 55 mm^2^. The dimensions of the 8 × 8 array is given in Table 2. Again, it was important to ensure the length of the feedlines is made such that there is phase coherency and power parity at the individual antennas constituting the 32-element array. A quarter-wave transformer is used with the power divider to ensure impedance matching at the input.

The 8 × 16 array was extended to 8 × 32 array using the above principles, as shown in Fig. 3b. The overall size of the array is 30 × 110 mm^2^. The dimensions of the F-parameters annotated in Fig. 3b are given in Table 3.

**Table 3:** Dimensions of the F parameters in the 8 × 32 antenna array.

Parameter	Length (mm)	Width (mm)
F1	1	1.4
F2	1	1.5
F3	0.5	1.5
F4	0.5	1.5
F5	0.5	1.1
F6	0.4	1.4

## Results and discussion

### Scattering parameter

**Figure 4:**
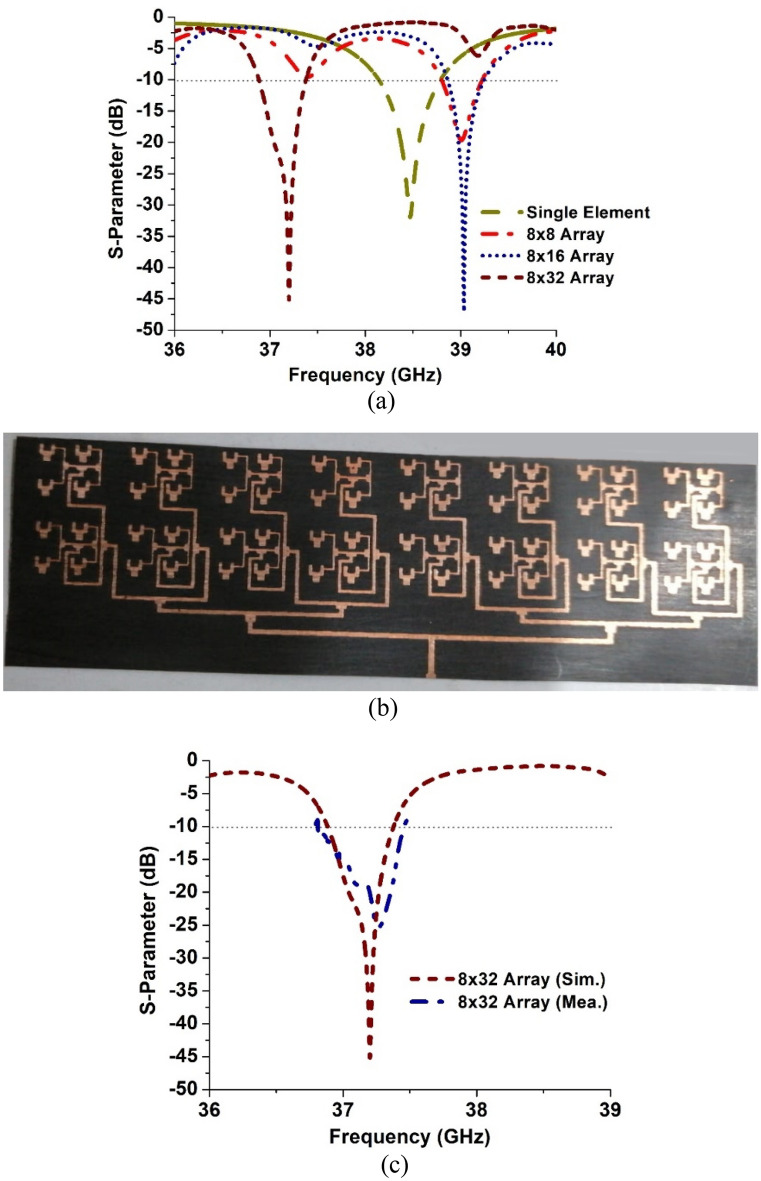
Reflection coefficient comparison of the three antenna array designs, (**a**) simulated, (**b**) photograph of the fabricated 8 × 32 array, and (**c**) measured results of the 8 × 32 array.

The simulated reflection coefficient of the single element antenna and the three antenna arrays, i.e., 8 × 8, 8 × 16 and 8 × 32, are shown in Fig. 4a. The single element antenna resonates at approximately 38.5 GHz with a reflection coefficient better than − 30 dB. The single element was used to construct the three arrays of increasing matrix size. Figure 4a shows the 8 × 8 array to resonate at 39 GHz with a reflection coefficient of − 20 dB, which is a reduction of nearly 10 dB compared to the single element antenna. The 8 × 16 array however has a significantly improved reflection coefficient of − 47 dB and it resonates at 39.1 GHz. This indicates excellent impedance matching. The 8 × 32 array too has a very good reflection coefficient of − 45 dB and it resonates at 37.2 GHz. In terms of footprint size, the 8 × 8 array occupies an area of 25.5 × 27.5 mm^2^ however this increases to 30 × 110 mm^2^ for a 8 × 32 array. The 8 × 32 operates at the 5G mm-Wave band. The fabricated prototype of the 8 × 32 array is shown in Fig. 4b. The measured reflection coefficient of the 8 × 32 array is shown in Fig. 4c. The measured result is in good agreement with the simulated one in terms of resonant frequency however the reduction in the reflection coefficient is attributed to unaccounted conductor loss, dielectric loss, and radiation loss. It should be noted that the conductivity of the metal, the skin effect, and surface roughness affect conductor losses in microstrip lines.

**Figure 5:**
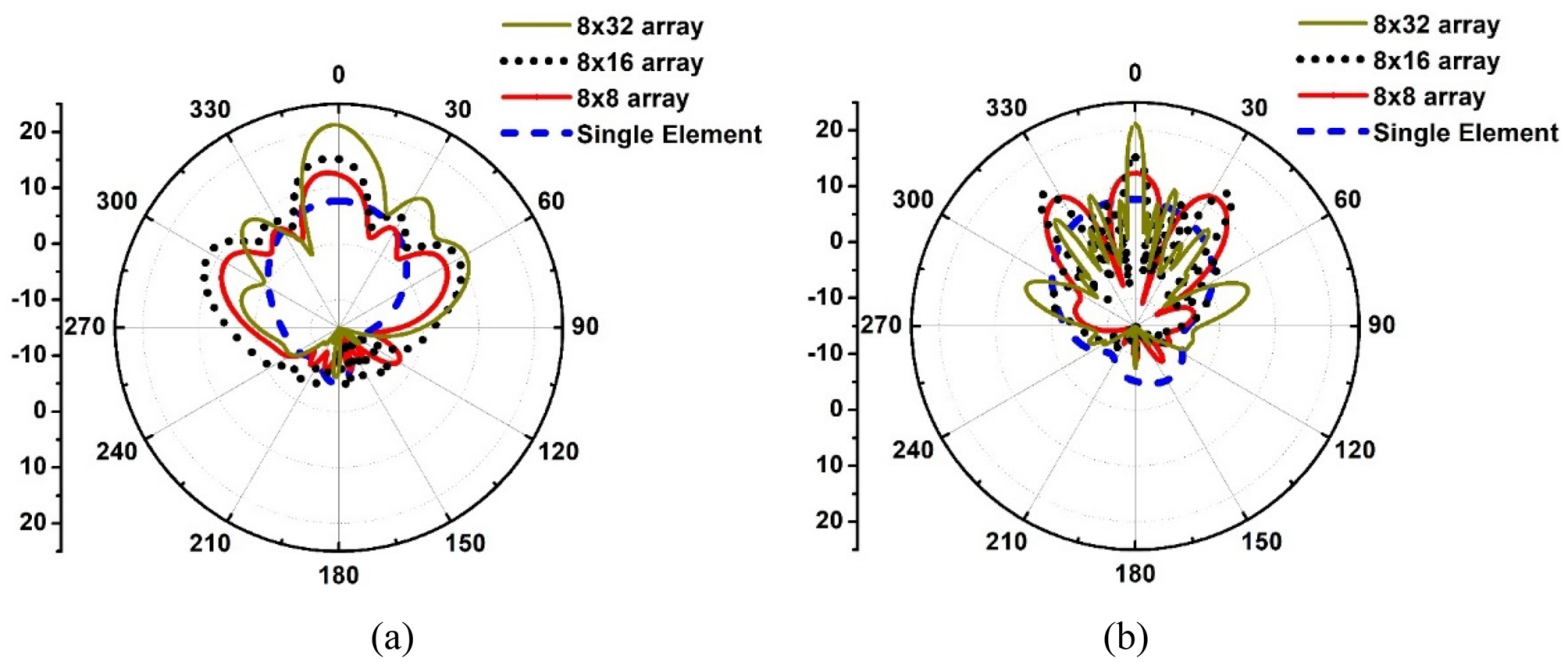
Polar radiation patterns of the single antenna element and the three arrays (8 × 8, 8 × 16, and 8 × 32), (**a**) phi = 0 degree plane, and (**b**) phi = 90 degree plane.

The radiation patterns of the proposed antenna and the three arrays of different matrix size using the antenn are compared in Fig. 5. The gain of single element antenna is 7.6 dBi. This gain is acceptable for a mobile handset but is not enough for 5G mm-Wave base stations that need to overcome signal attenuation resulting from path loss, multipath effects and atmoshoeric absorption. Figure 5 shows 8 × 8 array has a gain of 13 dBi however its side lobes are only less than − 6 dB in the phi = 0 degree plane however in the phi = 90 degree plane it is less than − 3 dB. This is not good as the system is prone to interference. In the case of the 8 × 16 array, it provides a gain of 15.3 dBi, which is acceptable for base stations however its side lobes too are less than − 6 dB in the phi = 0 degree plane and less than − 4 dB in the phi = 90 degree plane. In the case of the 8 × 32 array, it provides a substantial gain of 21.2 dBi and its side lobes are less than − 10 dB in the phi = 0 degree plane and less than − 12 dB in the phi = 90 degree plane, which is acceptable for practical applications. From Fig. 5 we can determine the angular width of the antenna in the phi = 90 degree plane. For the single antenna element the angular width is 82.5 degrees, for 8 × 8 array it is 18.4 degrees, for 8 × 16 array it is 7.2 degrees, and for 8 × 32 array it is 4.1 degrees. As expected the radiation beam becomes narrow and directed with increasing array size. The main purpose of this study was to verify the feasibility of the proposed antenna in arrays. However, further works is required to reduce the side lobes and to create a dual polarized array.

## Comparison with recent work

Over recent years numerous mm-wave antenna array designs have been investigated. Table 4 shows comparison of the proposed antenna array with recently reported works. In^21^ a dual band 5G multiple input multiple output (MIMO) antenna is reported that operates at 28 GHz and 38 GHz. The sub-array in^21^ consists of four gap-coupled microstrip slotted dual band patch antennas with corporate microstrip feed network. This sub-array exhibits a realized gain of 12 dBi at 28 GHz and 13 dB at 38 GHz. The antenna array in^22^ is based on stacked square ring patch arrangement. This antenna has a sharp roll-off and a filter like response between the operating bands due to the strongly coupled resonators. The antenna operates from 24.25 to 29.5 GHz and 37 to 40 GHz. This antenna array has a realized gain of between 5 and 6 dBi. In^23^ the authors have created an antenna array by stacking multiple split-ring resonators that interact with each other via aperture coupling. This antenna array operates from 24.25 to 29.5 GHz and has a realized gain between 5 to 6 dBi.

**Table 4:** Comparison of the proposed antenna array with recent works.

Refs	No. of elements	Center Freq. (GHz)	Gain (dBi)	Substrate permittivity (*ε*_*r*_)	Dimensions (mm)
^23^	1 × 2	26.87	~ 5–6	3.35	5 × 5
^22^	1 × 4	26.87	~ 5–6	3.35	5 × 5
^22^	1 × 4	38.5	~ 5–6	3.35	5 × 5
^27^	1 × 8	33.5	14.10	2.2	58 × 5.5
^28^	1 × 8	36	16.90	2.2	50 × 40
^24^	2 × 2	37	12.80	2.2	20 × 8
^25^	2 × 2	29	14.03	2.2	9.7 × 9.7
^21^	4 × 3	28	12.60	2.2	122 × 156
^21^	4 × 3	38	13	2.2	122 × 156
^26^	4 × 4	30.1	11.35	2.94	90 × 54
^29^	8 × 8	26	23.96	–	–
^30^	8 × 8	39	22	3.3	100 × 90
^31^	8 × 8	28	22	2.55	100.6 × 96.2
^32^	8 × 32	35	25.70	2.22	212 × 56
This work	8 × 32	37.2	21.20	2.20	110 × 30

The MIMO antenna array in^24^ consists of a two-antenna array combination where each antenna array consists of four elements which are arranged orthogonally with respect to each other. The antenna array has a gain of 12.8 dBi at 37 GHz. The 29 GHz magneto electric (ME) dipole array in^26^ is realized by the method of connecting and cutting patches. In^27^ a printed ridge gap waveguide 4 × 4 Butler matrix is shown to operate over the frequency range from 26.9 to 33.3 GHz with gain variation from 10.2 to 11.35 dBi. The array element in the broadband end-fire antenna in^28^ consists of a horizontally oriented printed electric dipole and a vertically aligned tapered slot radiator, where the two orthogonal radiated electric-field components are excited simultaneously. A parasitic director is introduced near the printed dipole to compensate for the gain degradation of the dipole element at higher frequencies.

In^29^ the 8 × 8 antenna array consists of a block of metallic array antenna sitting directly on the top surface of a printed circuit board (pcb) with electrical connections between them. The metallic array antenna is a metallic structure with 64 units of antenna radiating elements surrounded by a ring of metallic wall. The pcb provides the feeding mechanism for all 64 units of antenna radiating elements on the metallic array antenna. This antenna has a gain of 23.95 dBi at 26 GHz. The 8 × 8 element antenna array reported in^30^ uses a stacked multilayered pcb. It uses 16 commercial quad-core transmitter and receiver integrated circuits to independently control the phase and amplitude at each radiating element. The array designed at 39 GHz frequency band has a gain of 22 dBi. Reported in^31^ is an 8 × 8 aperture-coupled microstrip patch antenna array. It constructed from two substrate layers that are separated with an air gap. The patch antenna on the top layer is excited through a ground slot implemented on the top of the bottom substrate layer with a feedline on the bottom layer. This array is designed to operate over the 22–27 GHz frequency band with 22 dBi gain.

The work reported on 8 × 32 patch antenna arrays is scarce however the authors managed to find one paper^32^. The 8 × 32 antenna array in^32^ consists of a square patch antenna designed to operate at 35 GHz. The antenna array is reported to have a gain of 25.7 dBi. Although the gain in^32^ is higher than the proposed 8 × 32 antenna array however it has a much larger size by a factor of 3.6.

## Conclusion

Results presented in the paper show the feasibility of a novel patch antenna configuration in the design of high-gain antenna arrays for application in future 5G mm-Wave base stations. The gain provided by the proposed singular antenna is 7.6 dBi. Although, this gain is suitable for mobile communication devices but it is not sufficient to overcome path loss and atmospheric loss experienced my millimeter wave signals at the 5G base station. Hence, a 64-element array antenna was implemented using the proposed antenna. This antenna array is shown to have a bore side gain of 21.2 dBi at 37.2 GHz, an angular width of 4.1 degrees and side lobe levels less than − 10 dB. This antenna has a significantly smaller form factor than 8 × 32 antenna array reported to date.

## Declaration

All of the figures, materials, and data within the manuscript are original and owned by authors.

## Data Availability

All data generated or analyzed during this study are included in this article.
